# COVID-19 infection and cardiac angiosarcoma: a dangerous combination—a case report

**DOI:** 10.1186/s43057-021-00042-7

**Published:** 2021-02-24

**Authors:** Santiago A. Endara, Gerardo A. Dávalos, Gabriel A. Molina, Aldo B. Zavala, Patricia M. Ponton, Maribel Brito, Carlos Nieto, Vladimir E. Ullauri

**Affiliations:** 1 Department of Surgery Division of Cardiothoracic Surgery, Hospital Metropolitano, Av. Mariana de Jesús Oe7/47 y Conclina, Edificio Diagnóstico 2000 tercer piso 3/3, Quito, Ecuador; 2 Universidad San Francisco de Quito (USFQ) & Department of General Surgery, IESS Quito Sur, Quito, Ecuador; 3 PGY4 General Surgery P.U.C.E, Quito, Ecuador; 4 Department of Internal Medicine, Division of Pathology, Hospital Metropolitano, Quito, Ecuador; 5 Department of Internal Medicine, Division of Cardiology, Hospital Metropolitano, Quito, Ecuador

**Keywords:** COVID-19, Primary heart angiosarcoma, Heart tumor

## Abstract

**Background:**

The COVID-19 pandemic has strained all medical systems, especially in countries like Ecuador, where health services were already limited. These conditions, combined with a deadly and unusual disease, like primary heart angiosarcoma, can lead to severe outcomes. Angiosarcomas represent the most common and aggressive primary malignant heart tumor; regretfully, its clinical manifestations are vague and can be easily missed. Most patients become symptomatic when there is local invasion, embolization, or metastases, leading to late diagnosis and poor survival. High clinical awareness, adequate diagnosis, and prompt treatment are vital in these rare diseases, in which time is of paramount importance.

**Case presentation:**

We report the case of a 28-year-old female who had cough, hemoptysis, and ground-glass opacities in the CT (computed tomography). Since Ecuador is in the middle of this pandemic, she was misdiagnosed and mistreated. Primary heart angiosarcoma was diagnosed, and regretfully, the patient suffered multiple complications due to diagnosis and died.

**Conclusion:**

To this day, most cardiac angiosarcomas are found in a late-stage with distal metastasis and advanced local invasion. Sadly, this tumor is frequently missed due to its incidence and broad-spectrum of clinical symptoms. Considering that its manifestations can be misleading, misdiagnosis can occur, especially in pandemic times. Therefore, knowledge of other pathologies prevents COVID-19 from overshadowing other diagnoses, hence preventing delayed diagnosis or even misdiagnosis and consequent adverse outcomes for patients.

**Supplementary Information:**

The online version contains supplementary material available at 10.1186/s43057-021-00042-7.

## Background

Heart tumors are incredibly uncommon. Sarcomas are the most frequent primary malignant cardiac tumor, of which angiosarcoma is the most common and most aggressive histological entity [1, 2]. Due to its vague symptoms, rarity, early metastases, and resistance to chemoradiotherapy/radiotherapy, the short-term prognosis of angiosarcoma patients are regularly low [3]. Since these tumors are rare, they can be mistaken with many pathologies, including SARS-CoV-2 (severe acute respiratory syndrome coronavirus 2) who tends to manifest with ground-glass opacities, similar to those of angiosarcomas [1, 3, 4].

We present the case of a 28-year-old female patient with a primary heart angiosarcoma (AS). She was misdiagnosed and found in the final stages of her illness. After a biopsy, she suffered from severe complications from her condition and passed away.

This manuscript reporting adheres to CARE guidelines [5].

## Case presentation

Patient was a 28-year-old female non-smoker with a past surgical history of breast implants. She presented to the emergency room with a 3-week history of recurrent episodes of hemoptysis and cough; at first, the symptoms were mild, so the patient did not seek any medical attention; nonetheless, they became more intense over time and were accompanied by severe shortness of breath, due to this, she was admitted to a private clinic. Complementary exams, at the time of admission, revealed leukocytosis, neutrophilia, and a ground-glass opacification in the right upper lobe on chest X-rays. SARS-Cov-2 was suspected, yet the initial COVID-19 RT-PCR (real-time reverse transcription-polymerase chain reaction) test from the nasopharyngeal swab was negative. Regretfully, the medical team did not consider other diagnoses at that time, and she was misdiagnosed as community-acquired pneumonia. Oxygen and antibiotics were initiated, yet, despite treatment, and after a week, the patient lacked significant improvement. Suddenly, on the seventh day of hospitalization, she experienced dyspnea on exertion and endured massive hemoptysis that required a transfusion of three packed red blood cells as her hemoglobin lowered below 6mg/dl. After the patient was stable, a contrast-enhanced chest CT identified the same ground-glass pattern, now on both lungs, multiple peripheral pulmonary nodules, pericardial effusion. Transthoracic echocardiography (TTE) was requested immediately, revealing a large vegetation in the posterior leaflet of the tricuspid valve; consequently, infectious endocarditis was suspected.

Blood cultures were requested and identified a methicillin-resistant Staphylococcus aureus (MRSA) three days after this event. Nonetheless, her clinical condition continued to deteriorate. Due to the ground glass pattern observed in both lungs, another COVID-19 test was requested on the twelfth day of hospitalization; this time, the test was positive.

COVID-19 therapy, including azithromycin and corticosteroids, was about to be administered. Nonetheless, as her clinical course was unfavorable, she was transferred to our hospital.

On arrival, a conscious but dyspneic and tachypneic patient was encountered, her blood oxygen saturation was 74%; hence, high-flow oxygen therapy was started. Her blood pressure was normal and cardiac auscultation was unremarkable without any murmurs. On chest examination, decreased expansibility was detected along with diffuse crepitations and crackles.

**Fig. 1:**
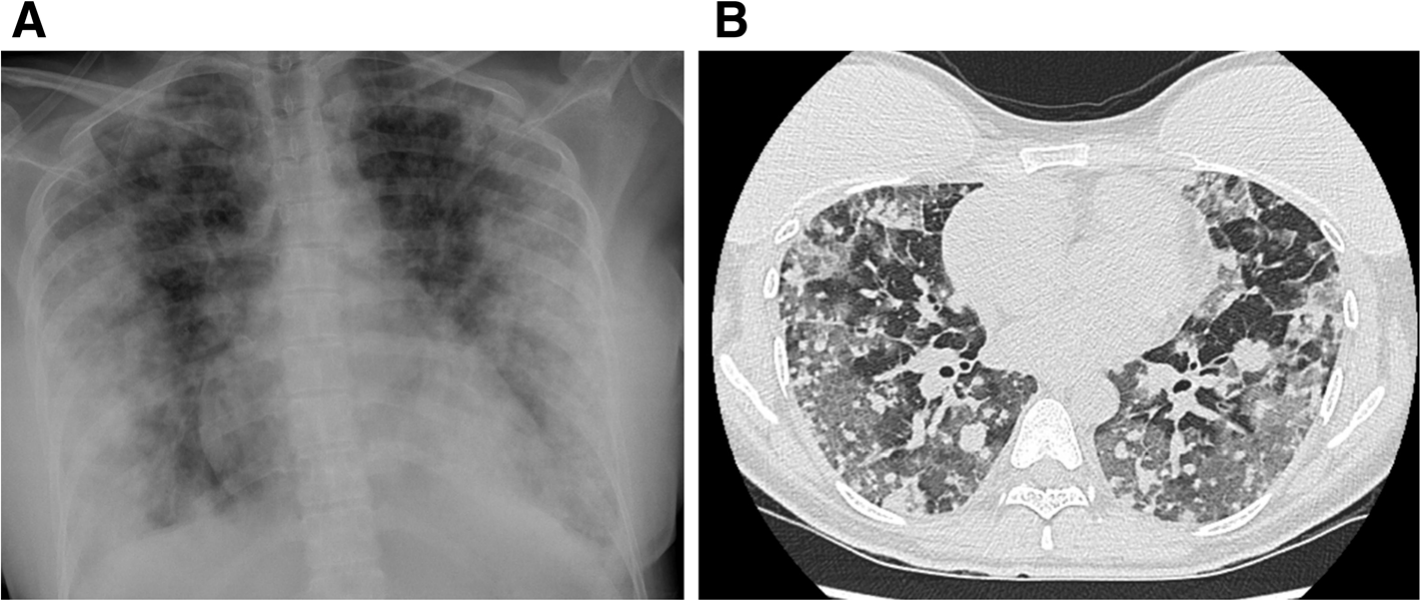
**a** Scout Image from Chest CT, revealing diffuse ground-glass opacification and pulmonary infiltrates. **b** Contrast-enhanced chest CT, presenting signs of diffuse alveolar hemorrhage and multiple pulmonary nodules

**Fig. 2:**
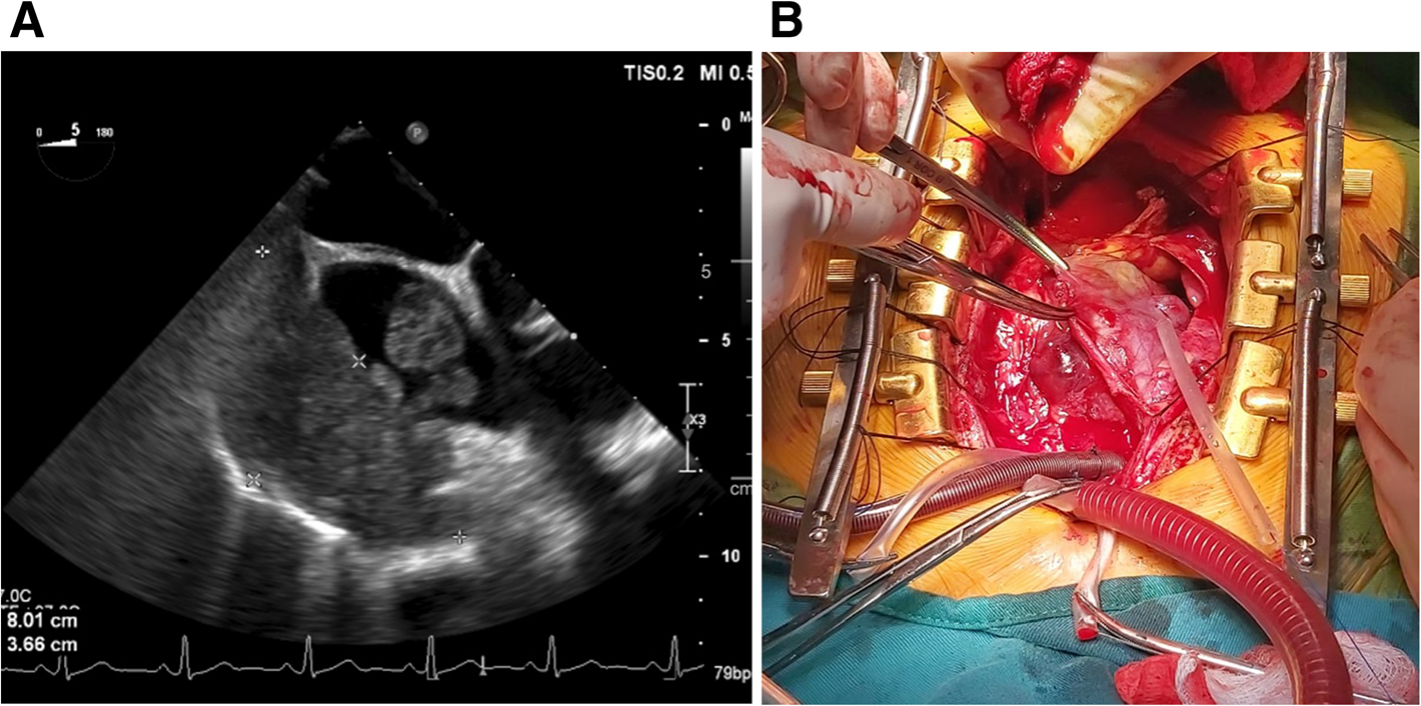
**a** TEE showing a hyperechoic mass in the right atrium. **b** Cardiopulmonary bypass, pericardiotomy showing tumor infiltration


Due to the discrepancy of the results in the COVID-19 tests, a new RT-PCR test was performed, which was negative. Also, as we only had the radiology reports but no images were available, new imaging studies were done and a new contrast-enhanced chest CT showed diffuse alveolar hemorrhage, the previously detected pulmonary nodules, and a right atrial mass (Fig. 1a, b). A new transesophageal echocardiogram (TEE) was done at our center, showing a large (7 × 6 × 4 cm) lobulated heterogeneous intracardiac mass within the right atrium; it had a broad base that reached the tricuspid valve. The mobile mass entered into the tricuspid orifice in diastole and back into the right atrium during systole. A widened inferior vena cava, mild tricuspid regurgitation, moderate pericardial effusion, and bilateral pleural effusions were also detected (Fig. 2a & Supplementary Video 1). With these findings, the medical team reached a definitive diagnosis. Although the pandemic has undeniably affected all our institutions and personnel, we should not lose awareness of the other pathologies that can harm our patients. Cardiac mass, multiple pulmonary nodules, hemoptysis are not the principal characteristics of SARS-Cov-2. Although ground-glass opacities can cloud our judgment in pandemic times, it should not define our practice as physicians. After a multidisciplinary meeting, the COVID-19 diagnosis was dismissed, and we focused on the cardiac mass.

**Additional file 1**: **Supplementary Video 1**: TEE showing a large (7 x 6 x 4 cm) lobulated heterogeneous intracardiac mass within the right atrium.

Cardiothoracic assessment was required, and wide resection surgery was decided. During surgery, and after a median sternotomy and cardiopulmonary bypass, the tumor was visualized. It had compromised the right atrial wall. It measured 6 × 6 × 4 cm and involved the atrioventricular sulcus; resection of the tumor was not possible as infiltration was extensive. Therefore, multiple biopsies were taken (Fig. 2b). Also, numerous pulmonary nodules were seen in the upper-middle and lower pulmonary lobes. Surgery was completed, and the patient was successfully weaned off cardiopulmonary bypass. Frozen section revealed an angiosarcoma (AS).

She remained stable during her first day in the ICU with ventilatory support; however, 28 hours after surgery, the patient underwent abrupt hypotension requiring a high dose of inotropes and vasopressors. As she remained unstable despite these measures, a new TEE revealed that the lobulated cardiac mass was absent, and only a small part of it was evident. (Supplementary Video 2). We also detected right ventricle dilation and dysfunction. Tumor embolism was presumed. Pulmonary angiogram and emergent embolectomy were considered; nonetheless, due to her condition and grim prognosis no other therapies could have improved her recovery. Regretfully the patient suffered an irreversible cardiogenic shock and died.


**Additional file 2: Supplementary Video 2**: TEE without the lobulated cardiac mass only a small part of it is evident.

**Fig. 3:**
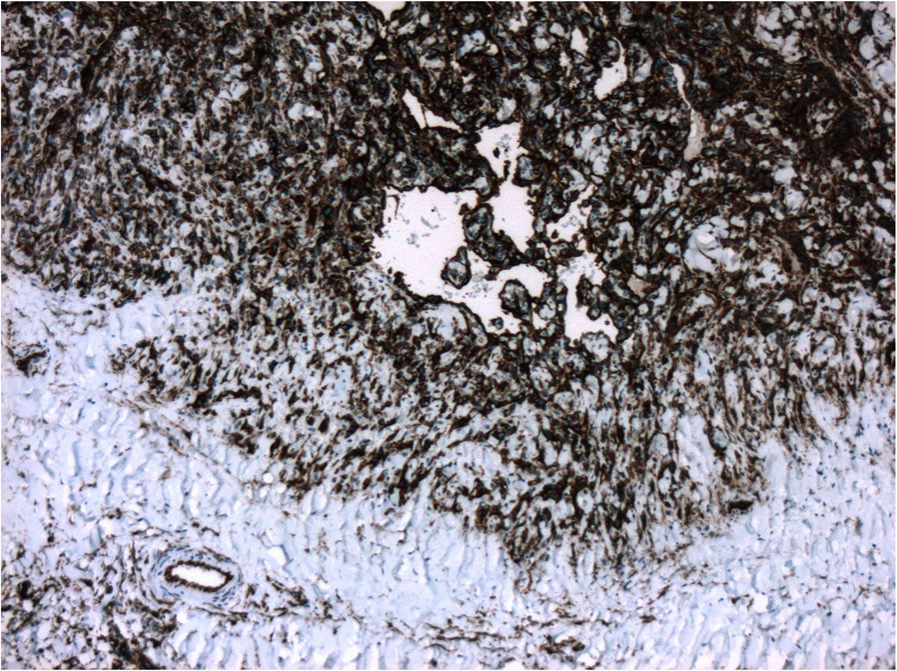
Immunohistochemistry positive for CD34

Pathology confirmed a high-grade primary heart angiosarcoma (AS), with multiple spindle and ovoid cells; immunohistochemistry was positive for CD34, vimentin, FLI-1, and CD31 (Fig. 3).

## Discussion

Cardiac tumors are a challenging and rare condition [2]. They account for 0.002% to 0.2% of all tumors in the adult population [1, 2]. Primary tumors of the heart are rarer, with incidences ranging from 0.001 to 0.03%. The majority of these tumors are benign (75%) [2–4]. Of the remaining malignant tumors (25%), cardiac sarcomas constitute 95% of the cases [3]. Primary cardiac angiosarcoma (AS) is the most common histological subtype (30%) and is characterized by its deadly nature [3, 4].

Since the first reports by Beck, Thatcher, and Zollicofferus in 1685, little progress has been made in improving survival outcomes [3, 6]. This is mostly due to its late diagnosis, high tumor invasiveness, inability to achieve complete resection and lack of response to the adjuvant therapy [2, 7]. They are thought to appear due to exposure to chemicals or synthesized materials; however, its etiology is still under research [3, 4]. Histologically, cardiac angiosarcomas consist of many well-differentiated vascular channels mixed with poorly differentiated solid areas of epithelioid cells and spindle cells [4, 7]. Immunohistochemistry is usually needed to confirm the diagnosis. These tumors show positivity for CD31, FLI-1, CD34, and von Willebrand factor [7, 8], as it was found in our patient.

Primary cardiac AS can occur at any age but is common among middle-aged men (30-40 years) and can appear anywhere in the heart [9]. They tend to appear mostly in the right atrium (90%) as a multicentric mass [3]. Regretfully, they are characterized by aggressive growth within the myocardial wall and can project into the atrial chamber. Due to this, they tend to metastasize to the lung, bone, spleen, and liver leading to a poorer prognosis (5-13 months survival rate) [6, 7].. When the lung is involved, the patient can present with multiples nodules and ground-glass opacities due to the gas retention in the peripheral alveoli as the alveolar walls thicken [7, 8], as it was found in our patient.

This can be mistaken with many pathologies, including SARS-CoV-2 (severe acute respiratory syndrome coronavirus 2) who first appeared at the beginning of December 2019 and tends to manifest on lung CT scans as bilateral, subpleural, ground-glass opacities with air bronchograms, ill-defined margins, and a slight predominance in the right lower lobe [10]. Ecuador has been hit hard by the pandemic since February 26, 2020. It is still trying to overcome this disease, and due to this, many patients who arrive with ground-glass opacities could be misdiagnosed [10, 11]. In our case, the patient presented with ground glass-opacities and a mass in the heart, nonetheless as Ecuador is in the middle of a pandemic, misdiagnosis occurred.

Although we need to be watchful in the diagnosis of COVID-19 in this pandemic period, we should not overlook the common and uncommon diseases [12]. Patients fitting the standard case presentation but without a SARS-CoV-2 infection may be misdiagnosed due to this availability bias [12, 13]. This may lead to delayed treatment of the actual etiology and an increased risk of adverse outcomes [13], as our case patient experienced. Throughout this pandemic and despite the varied presentations of COVID-19, it is essential that availability bias does not blind clinicians and lead to diagnostic oversight of other non-COVID-19 conditions [12], such as angiosarcoma. Clinicians must not ignore their traditional diagnostic processes and continue to consider various differential diagnoses during this and any other pandemics [12, 14].

Diagnosis for primary heart AS is challenging as these tumors have non-specific symptoms, including chest pain, vomiting, cough, hemoptysis, shortness of breath, and fatigue [3, 4]. Some can even develop arrhythmias, syncope, pericardial effusion, and cardiac tamponade. These symptoms will be dependent on the extent of infiltration and the extent of metastases [6, 7]. Pulmonary tumor embolism syndrome has been reported to be found in 3% to 26% of autopsies conducted on patients with solid tumors; it can present in a variety of ways, from asymptomatic to severe cases with hemodynamic instability resulting in death [15, 16]. Sarcomas have an increased propensity for emboli development, and when it occurs, overall mortality is high (64%) [16, 17]. Unfortunately, this event happened to our patient. We suspect massive tumor embolus caused severe pulmonary hypertension that led to heart failure and death, as the TEE revealed that the lobulated cardiac mass was absent. Only a small part of it was evident, along with right ventricle dilation and dysfunction.

Early diagnosis in primary heart sarcomas is still tricky as many patients will remain asymptomatic until the tumor has grown to a specific size or has developed regional spread [6], as it happened to our patient. Imaging studies are essential in the diagnosis and prognosis [3, 4]. CT, echocardiography, and PET/CT (positron emission tomography-computed tomography) can help; nonetheless, cardiac MRI (magnetic resonance imaging) is the gold standard. It gives information on the infiltration of the myocardium [6]. Treatment is based on a multimodal approach; wide resection surgery, high dose chemotherapy, and radiotherapy combined seem to give patients better survival rates (12 to 30 months) [7, 8]. However, the inability to achieve complete resection and poor outcomes associated with orthotopic heart transplantation still make primary cardiac angiosarcomas a deadly disease [3, 9].

The entire ramifications of this pandemic are yet to be seen, and a unique awareness of atypical presentations allows SARS-CoV-2 to be a differential so that it can be appropriately investigated [18]. Knowledge of other pathologies prevents COVID-19 from overshadowing other diagnoses, hence preventing delayed diagnosis or even misdiagnosis and consequent adverse outcomes for patients “in a time of zebras, don’t forget about the horses” [12].

## Conclusions

Cardiac angiosarcoma is frequently missed due to its incidence and broad-spectrum of clinical symptoms. Considering that its manifestations can be misleading, misdiagnosis can occur, and this, combined with the tumor aggressiveness, will usually lead to a grim outcome. Coordinated action from a multidisciplinary team is required to try to overcome this fatal disease. Until further studies can provide insight into the tumor behavior and evolution, early surgery is the patient’s best chance for prolonging survival; therefore, high clinical suspicion and early diagnosis, even during pandemic times, are of paramount importance.

## Data Availability

All data and materials have been approved by all authors.

## Abbreviations

CT: Computed tomography
SARS-Cov-2: Severe acute respiratory syndrome coronavirus 2
RT-PCR: Reverse transcription polymerase chain reaction
TEE: Transthoracic echocardiography
MRSA: Methicillin-resistant Staphylococcus aureus
ECG: Electrocardiogram
ICU: Intensive care unit
MRI: Nuclear magnetic resonance imaging
AS: Angiosarcoma
